# COVID-19 related epigenetic changes and atopic dermatitis: An exploratory analysis

**DOI:** 10.1016/j.waojou.2024.101022

**Published:** 2025-01-07

**Authors:** Zhenwei Tang, Yu Chen, Yuzhen Ouyang, Yu Peng, Xiaoyong Man

**Affiliations:** a Department of Dermatology, Second Affiliated Hospital, Zhejiang University School of Medicine, Hangzhou, China; b Clinical Medicine Eight-year Program, Xiangya Hospital, Central South University, Changsha, China; c Department of Rheumatology, Second Affiliated Hospital, Zhejiang University School of Medicine, Hangzhou, China

**Keywords:** COVID-19, Atopic dermatitis, DNA methylations, LMAN2 protein, Epigenetics

## Abstract

**Background:**

While epidemiological data suggest a connection between atopic dermatitis (AD) and COVID-19, the molecular mechanisms underlying this relationship remain unclear.

**Objective:**

To investigate whether COVID-19-related CpGs may contribute to AD development and whether this association is mediated through the regulation of specific genes’ expression.

**Methods:**

We combined Mendelian randomization and transcriptome analysis for data-driven explorations.

**Results:**

Among the 172 CpGs -associated with COVID-19 infection, merely 3 of them exhibited significant impacts on the risk of AD, including cg04543273, cg11916609, and cg10636246. In the following analysis of the causal effects of CpGs and their related gene expression, cg04543273 inhibited LMAN2 expression. However, there was not a significant impact of cg11916609 and cg10636246 on the expression of their corresponding genes. Besides, transcriptome analysis suggested that LMAN2 expression was significantly upregulated among the COVID-19-infected population, and LMAN2 expression was obviously correlated with Type 2 helper cells across different post-infection time points.

**Conclusion:**

Overall, this study provides new insights of the COVID-19-related onset and exacerbation of AD-COVID-19-related epigenetic changes and their regulatory impact on transcription. A novel role of LMAN2 was proposed in the relationship between viral infection and AD. More studies are warranted to further explore the mechanism of LMAN2-related immunopathology.

## Introduction

Although it has been over 3 years since its first emergence, COVID-19 undoubtedly exerts huge impacts on human life, both biologically and mentally. Apart from the general post-covid syndromes such as fatigue and dyspnea,^1^ the emergence or aggravation of immune-mediated inflammatory diseases (IMIDs) associated with COVID-19 infection received increasing interest from researchers.^2^^,^^3^ A little evidence has suggested that the overactivation of immune response resulting from COVID-19 infection may be accountable for the following emergence of aggravation with IMIDs.^4^ Yet, it remained intriguing and vague how COVID-19 infection gives rise to the detrimental immune response and related diseases from a molecular perspective.

Atopic dermatitis (AD), is a chronic inflammatory dermatosis, that features a skew of T2 immune response and owes the highest disease burden among skin diseases.^5^ Similar to other IMIDs, AD can be easily triggered by infections.^6^ As for COVID-19, worsening and flare-ups of AD during the COVID-19 pandemic have been widely reported.^7^^,^^8^ On the other hand, elevated levels of T2 cytokines and T helper 2 (Th2) have been observed among the COVID-19-infected population.^9^^,^^10^ Despite the association between AD and COVID-19, results from a recent Mendelian randomization (MR) analysis revealed negative causal effects between AD and COVID-19, bidirectionally.^11^ Therefore, it is vital to figure out whether there is another explanation for the relationship between AD and COVID-19.

With the progression of COVID-19 research, the epigenetic effect of COVID-19 exposure has been described and its role in shaping the human immune system has been emphasized.^12^^,^^13^ Moreover, recent studies have also shed light on the contributory roles of aging and diseases of epigenetic changes related to COVID-19.^14^^,^^15^ Exploring COVID-19-related diseases from an epigenetic perspective may provide us with a novel understanding of infection and IMIDs.

Given the context provided above, we hypothesize that the association between AD and COVID-19 may stem from epigenetic modifications related to COVID-19, such as changes in DNA methylation. To overcome the lack of datasheets containing both information about COVID-19 and AD as well as potential bias related to environmental factors, we utilized a genetic proxied methods-MR analysis to detect the causal effects between COVID-19-related DNA methylation and AD.

## Methods

### Overall designing and datasheets

**Fig. 1:**
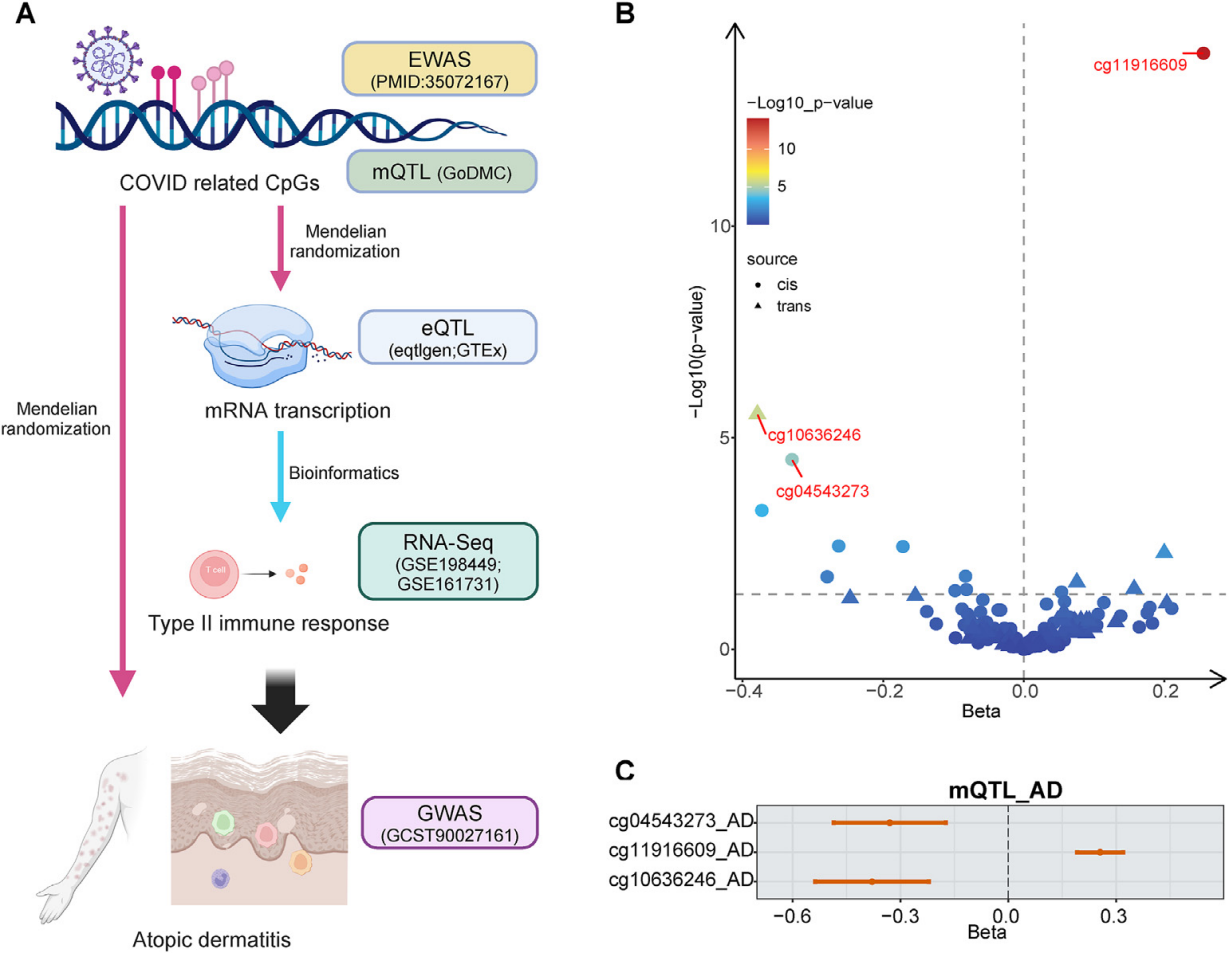
(A) The overall design of this study (Created in BioRender. Peng, Y. (2024) https://BioRender.com/j29j954). (B) The volcano plot shows the MR results of 172 CpGs associated with COVID-19 infection on AD. The dashed line indicates the significant threshold with P < 0.05. (C) The forest plot showed the detailed beta value of each CpG showing significant causality on AD

As is shown in Fig. 1A, the overall design of this study aimed to explore which COVID-19-related CpGs might promote the risk of AD, and whether these promotions rely on their regulation of certain genes’ expression. To achieve these goals, we applied MR, a well-established robust tool for causal inference. This method in casual inference depends on simply using genetic instrumental variables (IVs). The random allocation of genetic alleles during gamete formation is expected to reduce confounding in genetic associations and thus IVs made of genetic alleles are highly independent of potentially confounding environmental exposures. Besides, owing to the plentiful public GWAS data, it is greatly mature and convenient to conduct casual inference when RCTs are not feasible.^16^^,^^17^ Meanwhile, to explore the molecular and pathophysiological effects of COVID-19-related CpGs and their related genes, we applied bioinformatic analysis based on large-scale RNA-seq datasheets.

Detailed information about the datasheets we used is shown in Table S1. To identify the CpGs significantly associated with COVID-19 infection, we used the data from Epigenome-wide association analysis (EWAS) by Konigsberg et al,^18^ with a threshold of 1e-7. For AD genome-wide association studies (GWAS), we used the data from Sliz et al, where 3 large-scale biobank data were integrated and the heterogeneity of AD definition was considered.^19^ For quantitative trait locus (QTL) data, large-scale and reputable datasheets or databases were applied, respectively.^20^^,^^21^ To avoid interference from the ethics and genome reference version, all the data sheets we analyzed in this study were based on European ancestry and GRCh37.

For transcriptomic analysis, we used RNA-seq data of peripheral blood from GSE161731 and GSE198449. The former consists of data from 77 COVID-19-infected participants and 19 healthy participants, and is mainly utilized for the comparison between COVID-19 infection and healthy control. The latter is based on longer observation of 475 COVID-19-infected subjects, mainly utilized for the comparison of transcriptome changes at different time stages after infection.

### MR analysis

IVs were extracted from previous summary-level QTL data of exposure traits. For CpGs, their DNA methylation QTLs (mQTLs) were retrieved from http://mqtldb.godmc.org.uk/about, a large database collecting mQTL results from 36 cohorts of 27,750 European samples in whole blood.^20^ To explore causal associations between targeted genes’ expression and AD, 2 *cis*-eQTL (expression QTL) databases in blood tissue were used. For one thing, *cis*-eQTL information from the eQTLGen Consortium, where results from blood-derived expression from 31,684 individuals were provided.^21^ Moreover, *cis*-eQTL data from GTEx with a smaller sample size (N = 670) was also utilized for analysis.^22^ Because whole summary-level eQTL data from eQTLGen was not enough to support part of the MR analysis, only eQTL data from GTEx was applied to verify the CpG regulation effect on mRNA expression.

Due to different significance thresholds in mQTL data, we separately selected significant candidate IVs for MR analysis from *cis*-mQTL (±1 Mb from DNA methylation site; significant level: P < 1e-8) and *trans*-mQTL (>1 Mb from DNA methylation site; significant level: P < 1e-14) data as database suggests.^20^ As for eQTL data, candidate *cis*-eQTLs were defined with the same criteria, ±1 Mb from the center of the corresponding gene and P < 5e-8, in both eQTL databases. After filtering with significant level and minor allele frequency (>0.01), candidate IVs went through clumping with parameters r2 = 0.01 and kb = 10000 to guarantee the independence of IVs reducing the impact of linkage disequilibrium.

In the main MR analysis, 3 default methods including weighted median, inverse variance weighted (IVW), and MR Egger were utilized to evaluate the causal association. The wald ratio model was only applied for the single IV condition. Bonferroni corrections were applied to adjust the significance thresholds for the analysis. The IVW functioned as the main test in most cases, except for the number of IVs was limited when the Wald ratio was set as the main test, instead. Potential heterogeneity of MR analysis was detected using the Q tests of IVW and MR-Egger, while pleiotropy was detected using the MR-Egger intercept test and MR-PRESSO test.^23^ In addition, the directionality between the exposure and outcome was validated by the results of the MR-Steiger analysis, and weak instrument bias was calculated by F-statistic <10.^24^ The ethical methods of assessment, diagnostic criteria for diseases, committee approval, and participants' consent can be found in the original articles. R (4.2.0) packages “TwoSampleMR”^25^ and “MRPRESSO”^23^ were used for MR analysis. The significant P value was set as 0.05.

### Transcriptomic analysis

Processed RNA-seq data were retrieved and downloaded from the GEO database. For GSE161731, TPM data were downloaded and the different expression levels of LMAN2 among COVID-19-infected and healthy groups were compared using the Wilcoxon rank-sum test. For GSE198449, counts data were downloaded, processed, and transformed into TPM data using the R package “IOBR”.^26^ To describe the time-dependent variation of transcriptome after COVID-19 infection, we divided the samples into several time groups according to the sampling time after infection: Time group 0: 0 days after infection; time group 1: >0 and≤14 days; time group 2: >14 and≤28 days; time group 3: >28 and≤60 days; time group 4: >60 days; To gain insight on the relationship between LMAN2 and type 2 inflammation, a critical pathophysiological process of AD, we conducted Pearson correlation analysis between expression of LMAN2 and genes related to type 2 inflammation. Also, to test the association between LMAN2 expression and various immune cell levels, we calculated the levels of different immune cells using ssGSEA algorithms implemented in the R (4.2.0) package “IOBR”^26^ and performed Pearson correlation analysis subsequently. The significant P value was set as 0.05.

## Results

As is shown in Fig. 1B, among the 172 CpGs that were significantly associated with COVID-19 infection, merely 3 of them exhibited significant causal impacts on the risk of AD, including cg04543273, cg11916609, and cg10636246 (Detailed results are shown in Table S2). Among them, cg11916609 showed a positive impact on the risk of AD while cg04543273 and cg10636246 showed negative impacts, accordingly (Fig. 1C). Besides, the causal effects of these 3 CpGs on the risk of AD were unidirectional, according to the Steiger test (Table S3, All *P* value < 0.05).

**Fig. 2:**
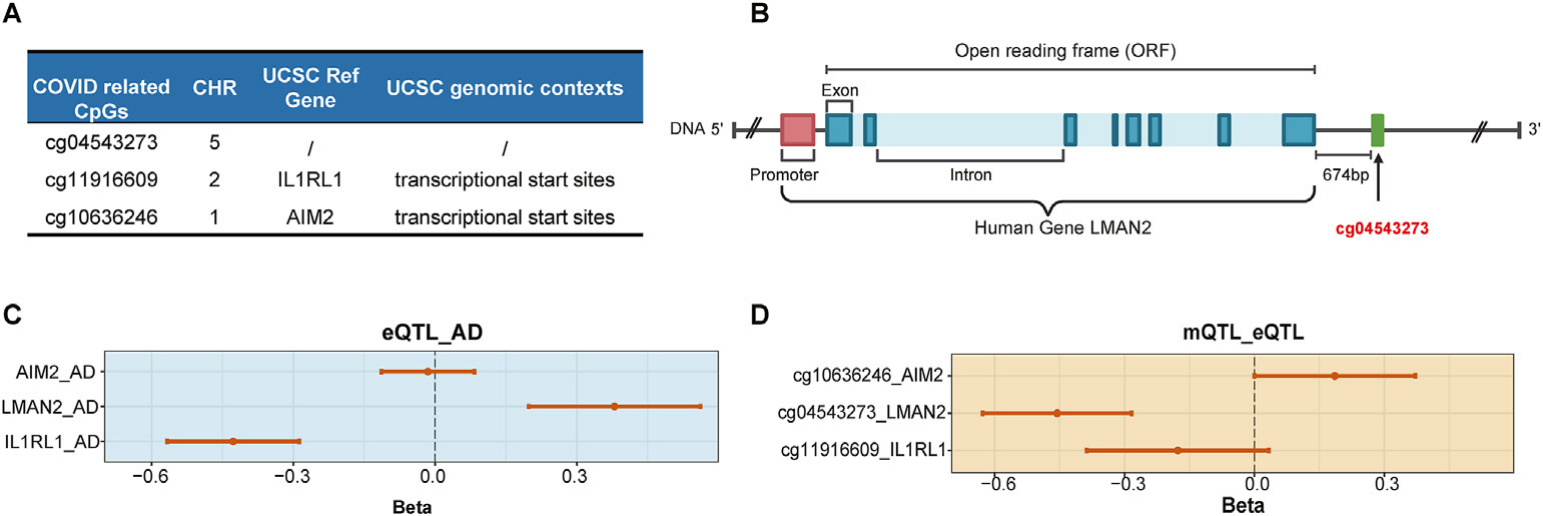
(A) Annotation information of the CpGs from the Infinium MethylationEPIC Array. (B) A schematic diagram illustrating the relative positional relationship between cg04543273 and the LMAN2 gene body (Created in BioRender. Peng, Y. (2024) https://BioRender.com/b34b047). (C) The forest plot showed eQTL MR analysis of the expression of related genes on AD. (D) The forest plot showed MR analysis of the effect of the CpGs on the expression of their related genes, respectively

To detect the genes regulated by these CpGs that might involve in AD, we retrieved the annotation information of the CpGs from the Infinium MethylationEPIC Array (Fig. 2A), and cg11916609 was annotated in the transcriptional start sites (TSS) of IL1RL1 while cg10636246 was annotated in the TSS of AIM2 (Fig. 2A). For cg04543273, as is shown in Fig. 2B and Fig. S1A, it is located 674bp downstream of the open reading frame (ORF) of LMAN2, and 19582 bp upstream of the ORF of MXD3. However, in the following MR analysis of eQTL, both AIM2 and MXD3 showed insignificant impacts on the risk of AD (Fig. 2C and Fig. S1B; Detailed results are shown in Tables S4–5). The expression of LMAN2 showed a significantly positive effect while IL1RL1 showed a significantly negative effect (Fig. 2C; Detailed results are shown in Tables S4–5). Since CpGs located in TSS are often repressive during transcriptional regulation,^27^ we suspected whether these CpGs exerted an effect on the risk of AD dependent on regulating corresponding gene expression.

In the following MR analysis on the causal effects of CpGs on their related gene expression, merely cg04543273 exhibited a negative effect on the expression of LMAN2 and MXD3 (Fig. 2D and Fig. S1B; Detailed results are shown in Tables S6–7), while the impact of cg11916609 and cg10636246 on the expression of related genes were insignificant (Fig. 2D; Detailed results are shown in Tables S6–7). Thus, the results from MR supported that cg04543273 and LMAN2 were involved in the association between 10.13039/100020014AD and COVID-19.

**Fig. 3:**
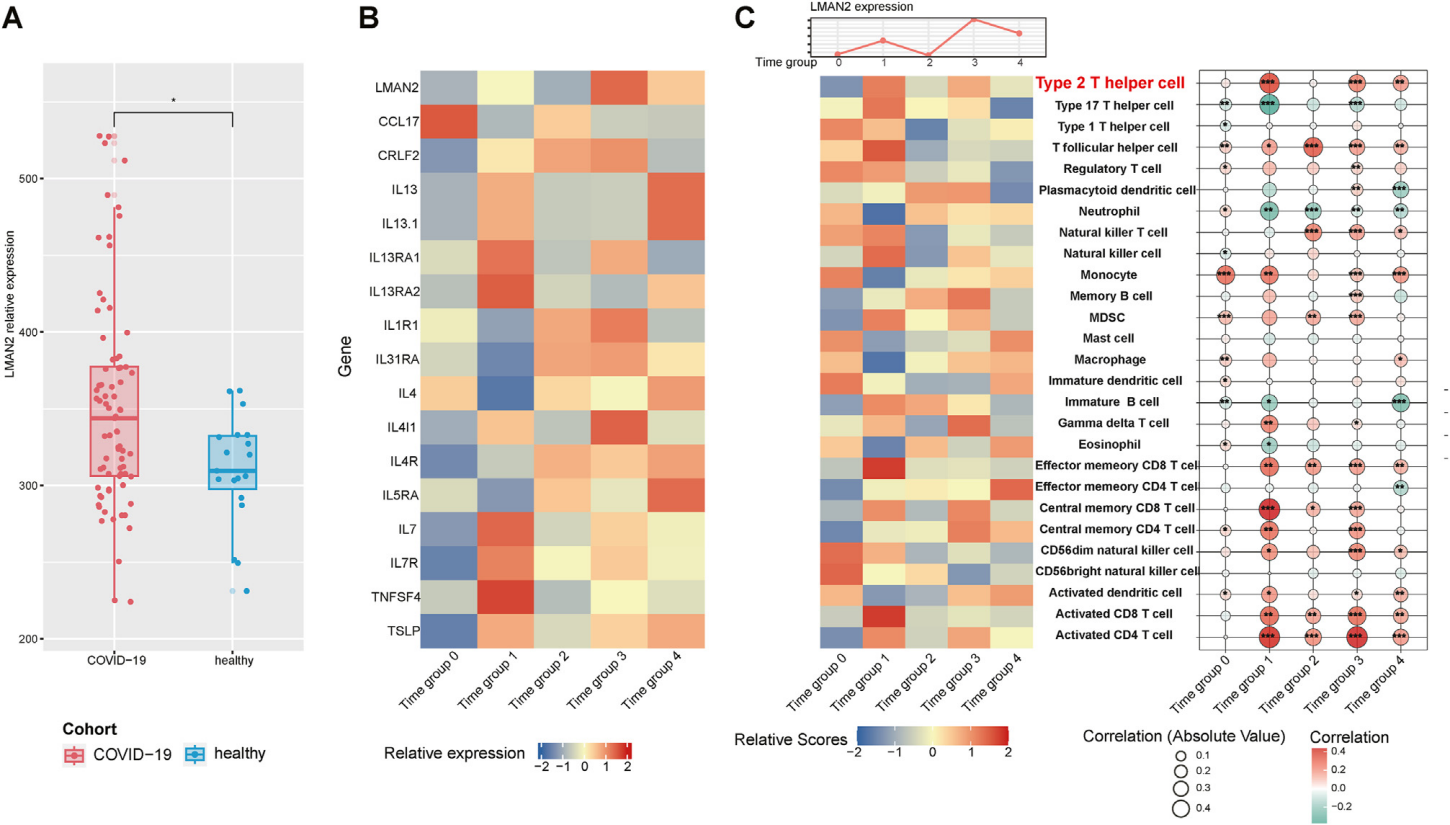
Bioinformatic analysis of RNA-seq data. (A) Expression of LMAN2 in COVID-19 infected and healthy groups. The p values were calculated using the Wilcoxon rank-sum test. (B) Heatmap shows the relative expression of LMAN2 and type 2 inflammation-related genes among different time groups post-COVID-19 infection (Time group 0: 0 days after infection; time group 1: >0 and≤14 days; time group 2: >14 and≤28 days; time group 3: >28 and≤60 days; time group 4: >60 days). (C) Related levels of different immune cells and their correlation with LMAN2 expression among different time groups

To further validate our findings, we first examined the transcriptome between the COVID-19-infected population and the healthy population. As illustrated in Fig. 3A, the expression of LMAN2 increased significantly among the COVID-19-infected population compared with the healthy population, while no significant difference was observed for MXD3 (Fig. S1C). Then, we analyzed the expression dynamics of LMAN2, type 2 inflammation-related genes, and related levels of immune cells among different time groups after COVID-19 infection (Fig. 3B–C), there were 2 peaks of LMAN2 expression during time group 1(>0 and≤14 days) and time group 3 (>28 and≤60 days), accordingly. In the first peak, IL13RA1, IL13RA2, and TNFSF4 were mostly upregulated while in the second peak, merely IL4I1 reached its peak although more genes are upregulated (Fig. 3B). As for the immune cells, the expression of LMAN2 was significantly correlated with Type 2 T helper cell among time group 1,3,4, but not significantly correlated with eosinophils or mast cells in most time groups (Fig. 3C).

## Discussion

The role of viral infections in the onset and exacerbation of IMIDs has been a critical topic for clinical immunology. With the release of COVID-19-related omics data, exploring this topic from the perspective of the association between COVID-19 and IMIDs became possible. In this study, we managed to discuss the association between COVID-19 and AD, based on large-scale GWAS and transcriptome data, combined with large-scale eQTL and mQTL data, with the help of MR and bioinformatics analysis. For the first time, our study explored the important role of the COVID-19-related epigenetic marker cg04543273 and its associated gene LMAN2 in the COVID-19-related onset and exacerbation of atopic dermatitis.

The LMAN2 gene encodes a type I transmembrane lectin that is involved in the transport shuttle function between the endoplasmic reticulum, Golgi apparatus, and plasma membrane, which are the keys to coronavirus assembly and budding.^28^ Moreover, its serum levels have been reported to be associated with inflammatory diseases like sepsis.^29^ Regarding its role in allergic diseases, Xu et al. integrated multiple large cohorts and found that CpG site cg15344640 in the LMAN2 gene region was significantly associated with the onset of childhood allergic diseases including AD.^30^ The latest GWAS study also proposed that the SNP site rs4532376 in the intronic region of LMAN2 is significantly associated with the onset of AD.^31^ However, the genomic sites proposed in the 2 aforementioned studies are mostly located within the LMAN2 gene's ORF, while the cg04543273 of interest in this study is located downstream of the LMAN2 gene's ORF, suggesting potentially different mechanisms of action. The focus of this study on LMAN2's role may be more specific to infection-related AD inflammation. However, undoubtedly, the study of the mechanisms of LMAN2 in AD holds great promise.

Another interesting finding in this study pertains to the levels of type 2 inflammation at different time points after COVID-19 infection. For COVID-19 infection, the levels of Th2 cells, similar to the expression of LMAN2, showed 2 peaks at 0–14 days and 28–60 days, with differences in the expression of type 2 immune molecules between these 2 peaks. In the first peak, the upregulation of molecules with accessible targeted drugs, such as IL13RA1, IL13RA2, and TNFSF4,^32^ was more significant, suggesting the practicality of early intervention with targeted drugs to prevent the occurrence or exacerbation of AD after COVID-19 infection. In the second peak, we observed the correlation of LMAN2 with a greater variety of immune cells, indicating that as a structural protein, its specific role in type 2 inflammation is likely to diminish with prolonged post-infection time. Therefore, indirectly proving that an early blockade of COVID-19 is necessary to prevent the promotion of AD.

This study has several strengths. First, we used MR for the causal inference of the effect of CPGs, and mRNA expression on the risk of AD, which avoids the interference from potential environmental confounders. Also, we repeatedly verified our findings using both cross-sectional and time-dependent transcriptomic data. However, the limitations of this study must be clarified. First, this study involved multiple datasheets, which might enlarge the sample size and lead to potential inconsistency in variable measurement and related bias. Second, since the current policy of COVID-19 control, it can be difficult to obtain trustworthy information on COVID-19 infection or enroll AD patients with initial infection of COVID-19. We could not provide any direct observation regarding the participants caught by both AD and COVID-19. Thirdly, this study is an explorative analysis merely focusing on human blood samples. Detailed mechanisms remained to be investigated using animal models. Lastly, as with most MR analyses about biomolecules, the impacts of cg04543273 and LMAN2 on the risk of AD should be carefully interpreted as they may not be decisive but contributory.

## Conclusion

Overall, our study reveals novel insights into how epigenetic changes and their regulatory impact on transcription displayed a novel sight of COVID-19-associated onset and exacerbation of AD. Meanwhile, LMAN2 was discovered as a potentially vital molecule elucidating the link between viral infection and IMIDs. These findings highlight LMAN2’ potential role in the pathogenesis of AD related to COVID-19. More studies are warranted to further explore the mechanism of LMAN2-related immunopathology.

## Abbreviations

IMIDs, immune-mediated inflammatory diseases; AD, atopic dermatitis; MR, Mendelian randomization; EWAS, Epigenome-wide association analysis; GWAS, genome-wide association studies; QTL, quantitative trait locus; IVs, instrument variables; mQTLs, DNA methylation QTLs; eQTLs, expression QTL; IVW, inverse variance weighted; ORF, open reading frame.

## Funding

This work was supported by the 10.13039/501100001809National Natural Science Foundation of China (82303997).

## Data availability statements

The original data sources can be found in Table S1. Processed data were can be obtained from corresponding author on appropriate request.

## Author contributions

All authors participated in the data collection, analysis and manuscript formation. Z.T. and Y.C conceptualized this study and drafted the manuscript. Z.T. and X.M. designed the study. Z.T., Y.C, Y.O. analyzed the data. Z.T., Y.C, Y.P. did the visualization. Z.T., and X.M. obtained the funding. All authors critically revised the manuscript, and gave final approval to the version submitted for publication. The corresponding author attests that all the listed authors meet authorship criteria and that no others meeting the criteria have been omitted.

Each author has participated sufficiently in the conception and design of the work or the analysis of the data, as well as the writing of the manuscript. All authors have read the manuscript and believe that the manuscript represents their valid work and are in agreement that the work is ready for submission to your journal and that they accept the responsibility for the manuscript's contents.

## Ethical statement

The data analyzed during the study comes from the relevant studies where written informed consent was received prior to participation.

## Declaration of competing interest

The authors declare no conflict of interest.

## Appendix A Supplementary data

Multimedia component 1 (A) A schematic diagram illustrating the relative positional relationship between cg04543273 and the gene bodies of LMAN2 and MXD3 (Created with Biorender (app.biorender.com)). (B) MR results of the causal effect of cg04543273 on MXD3 expression and MXD3 expression on AD. (C) Expression of MXD3 in COVID-19 infected and healthy groups. The p values were calculated using the Wilcoxon rank-sum test. Multimedia component 1
