# Supplementary figures and images for: COVID-19 related epigenetic changes and atopic dermatitis: An exploratory analysis
Source: World Allergy Organ J. 2025 Jan 7;18(1):101022. doi: 10.1016/j.waojou.2024.101022 (PMC11758953; doi:10.1016/j.waojou.2024.101022)

**A**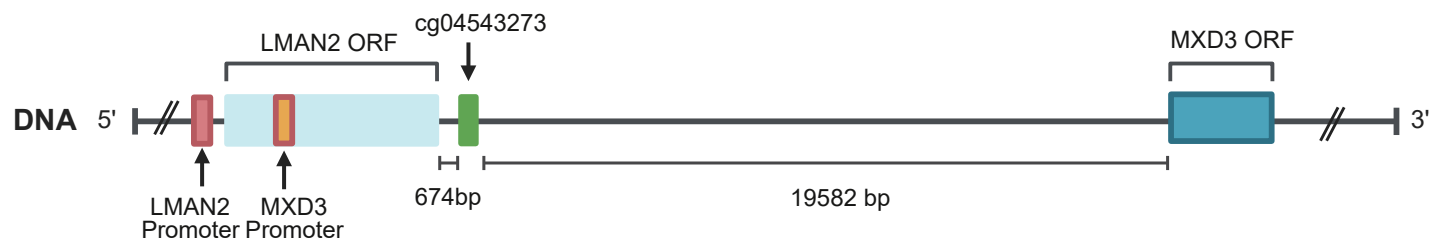**B**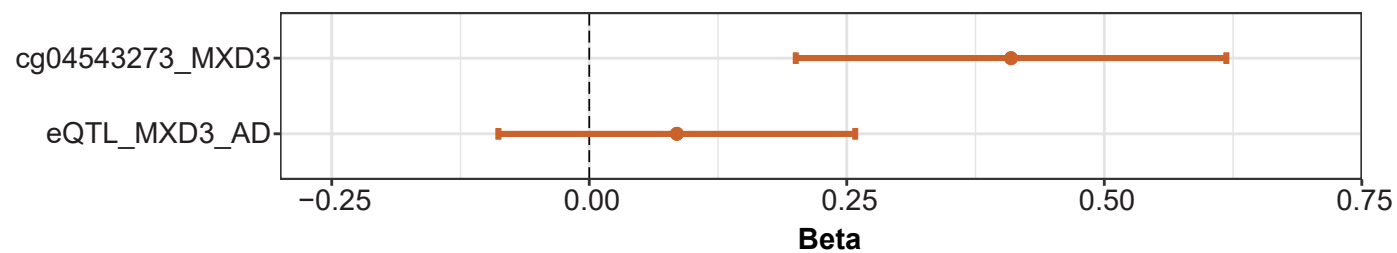**C**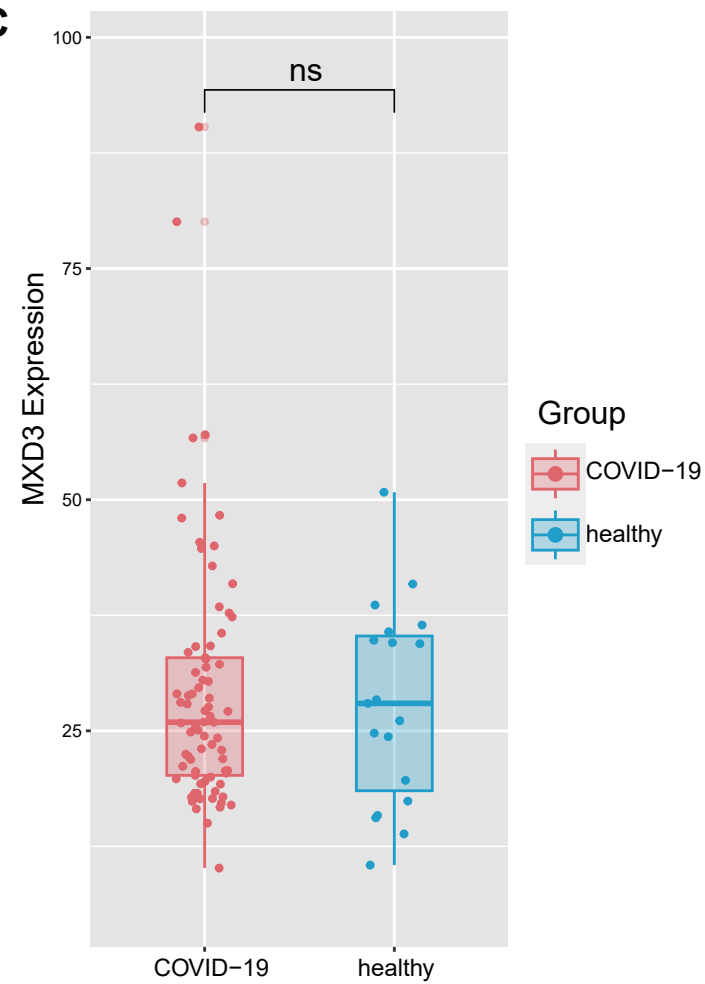

Supplement: Multimedia component 1 — (A) A schematic diagram illustrating the relative positional relationship between cg04543273 and the gene bodies of LMAN2 and MXD3 (Created with Biorender (app.biorender.com)). (B) MR results of the causal effect of cg04543273 on MXD3 expression and MXD3 expression on AD. (C) Expression of MXD3 in COVID-19 infected and healthy groups. The p values were calculated using the Wilcoxon rank-sum test. [file mmc1.pdf]
