# Supplementary material for: COVID-19 related epigenetic changes and atopic dermatitis: An exploratory analysis
Source: World Allergy Organ J. 2025 Jan 7;18(1):101022. doi: 10.1016/j.waojou.2024.101022 (PMC11758953; doi:10.1016/j.waojou.2024.101022)
Supplement: Multimedia component 2 [file mmc2.pdf]

Table S1: Detailed information on the datasheets used in this study.

data	web page	pmid
mqtl	http://mqtldb.godmc.org.uk/downloads	34493871
egtl1	https://eqtlgen.org/cis-eqtls.html	34475573
egtl2	https://www.gtexportal.org/home/downloads/adult-gtex/qtl	32913098
AD-gwas	https://www.ebi.ac.uk/gwas/studies/GCST90027161	34454985
EWAS_covid19_mqtl	https://doi.org/10.1038/s43856-021-00042-y	35072167

Table S2: MR results of 172 CpGs associated with COVID-19 infection on AD.

exp	out	nsnp	IVW	IVW_P	MRegger	MRegger P	weighted_ median	weighted_me dian_P	Wald_ratio	Wald_ratio P	type
cg02650017	AD	1	NA	NA	NA	NA	NA	NA	-3.73E-01	5.17E-04	cis
cg04543273	AD	1	NA	NA	NA	NA	NA	NA	-3.30E-01	3.28E-05	cis
cg05411199	AD	1	NA	NA	NA	NA	NA	NA	-8.17E-02	3.86E-02	cis
cg07477602	AD	3	5.37E-02	4.42E-02	2.12E-01	4.48E-01	5.48E-02	4.88E-02	NA	NA	cis
cg08398132	AD	2	-2.80E-01	1.93E-02	NA	NA	NA	NA	NA	NA	cis
cg09322555	AD	4	-9.77E-02	4.10E-02	-9.38E-02	7.70E-01	-9.52E-02	1.23E-03	NA	NA	cis
cg11916609	AD	2	2.56E-01	8.05E-15	NA	NA	NA	NA	NA	NA	cis
cg20692268	AD	1	NA	NA	NA	NA	NA	NA	-8.29E-02	1.87E-02	cis
cg21549285	AD	6	4.28E-02	2.37E-01	1.17E-01	1.01E-01	6.57E-02	3.36E-02	NA	NA	cis
cg24772388	AD	2	-1.72E-01	3.71E-03	NA	NA	NA	NA	NA	NA	cis
cg26312951	AD	1	NA	NA	NA	NA	NA	NA	-2.63E-01	3.59E-03	cis
cg00343127	AD	1	NA	NA	NA	NA	NA	NA	-3.45E-02	3.83E-01	cis
cg00388871	AD	3	3.21E-02	4.63E-01	1.91E-01	5.92E-01	5.01E-02	3.13E-01	NA	NA	cis
cg00421164	AD	1	NA	NA	NA	NA	NA	NA	3.82E-03	9.25E-01	cis
cg00533183	AD	2	5.74E-02	6.10E-01	NA	NA	NA	NA	NA	NA	cis
cg00590152	AD	4	2.00E-02	5.54E-01	-2.13E-01	4.36E-01	1.23E-03	9.74E-01	NA	NA	cis
cg00598235	AD	2	1.29E-02	7.71E-01	NA	NA	NA	NA	NA	NA	cis

cg01004980	AD	1	NA	NA	NA	NA	NA	NA	-9.71E-02	5.37E-01	cis
cg01028142	AD	1	NA	NA	NA	NA	NA	NA	5.44E-03	9.54E-01	cis
cg01459052	AD	1	NA	NA	NA	NA	NA	NA	-1.35E-02	7.51E-01	cis
cg02364826	AD	3	-3.65E-02	1.17E-01	-2.53E-02	6.11E-01	-3.45E-02	1.51E-01	NA	NA	cis
cg02481950	AD	3	1.40E-02	6.36E-01	-2.30E-02	8.08E-01	1.08E-02	7.25E-01	NA	NA	cis
cg02583484	AD	2	-4.20E-02	4.51E-01	NA	NA	NA	NA	NA	NA	cis
cg02741985	AD	5	-1.07E-03	9.63E-01	1.10E-02	7.99E-01	-1.71E-03	9.46E-01	NA	NA	cis
cg02744705	AD	2	8.19E-03	9.09E-01	NA	NA	NA	NA	NA	NA	cis
cg03234777	AD	5	3.23E-02	8.49E-02	1.66E-02	7.23E-01	2.05E-02	3.56E-01	NA	NA	cis
cg03290827	AD	4	9.25E-03	7.03E-01	1.09E-01	3.73E-01	1.14E-02	6.69E-01	NA	NA	cis
cg03398461	AD	1	NA	NA	NA	NA	NA	NA	7.46E-02	2.59E-01	cis
cg03663120	AD	9	2.09E-03	9.29E-01	2.55E-02	6.06E-01	-2.12E-02	4.27E-01	NA	NA	cis
cg03699074	AD	1	NA	NA	NA	NA	NA	NA	1.99E-02	7.39E-01	cis
cg03753191	AD	2	5.55E-02	4.56E-01	NA	NA	NA	NA	NA	NA	cis
cg03781224	AD	1	NA	NA	NA	NA	NA	NA	-6.16E-02	1.31E-01	cis
cg03887528	AD	2	-3.57E-04	9.87E-01	NA	NA	NA	NA	NA	NA	cis
cg04248312	AD	5	-1.05E-02	5.51E-01	-2.87E-02	4.80E-01	-1.27E-02	4.82E-01	NA	NA	cis
cg04306926	AD	1	NA	NA	NA	NA	NA	NA	-3.64E-02	4.61E-01	cis

cg04332373	AD	1	NA	NA	NA	NA	NA	NA	-2.23E-02	7.82E-01	cis
cg04445427	AD	4	-3.77E-03	8.93E-01	-6.02E-02	7.02E-01	3.24E-03	9.13E-01	NA	NA	cis
cg04571130	AD	3	1.52E-03	9.88E-01	-6.33E-01	4.60E-01	-8.05E-02	2.79E-01	NA	NA	cis
cg04577162	AD	2	9.65E-03	8.75E-01	NA	NA	NA	NA	NA	NA	cis
cg04871807	AD	5	-5.83E-03	7.15E-01	1.19E-02	6.23E-01	-2.29E-03	8.89E-01	NA	NA	cis
cg04955573	AD	4	2.51E-02	6.43E-01	1.07E-01	4.17E-01	3.60E-02	5.61E-01	NA	NA	cis
cg05316065	AD	1	NA	NA	NA	NA	NA	NA	-1.74E-02	8.68E-01	cis
cg05609160	AD	6	6.46E-03	6.75E-01	1.10E-03	9.64E-01	7.04E-03	6.55E-01	NA	NA	cis
cg05663031	AD	2	1.21E-02	6.38E-01	NA	NA	NA	NA	NA	NA	cis
cg05767306	AD	2	6.87E-03	6.73E-01	NA	NA	NA	NA	NA	NA	cis
cg05778424	AD	1	NA	NA	NA	NA	NA	NA	-4.33E-02	3.59E-01	cis
cg05934015	AD	1	NA	NA	NA	NA	NA	NA	1.38E-01	1.62E-01	cis
cg05955436	AD	6	1.46E-02	5.14E-01	-1.43E-02	7.81E-01	7.28E-03	7.55E-01	NA	NA	cis
cg06043201	AD	1	NA	NA	NA	NA	NA	NA	1.64E-01	2.97E-01	cis
cg06070445	AD	1	NA	NA	NA	NA	NA	NA	1.67E-03	9.63E-01	cis
cg06112967	AD	1	NA	NA	NA	NA	NA	NA	-7.12E-04	9.92E-01	cis
cg06146977	AD	2	1.06E-01	1.48E-01	NA	NA	NA	NA	NA	NA	cis
cg06850597	AD	1	NA	NA	NA	NA	NA	NA	-1.38E-01	1.28E-01	cis

cg06981309	AD	6	1.45E-02	5.52E-01	1.67E-02	7.47E-01	9.85E-03	6.74E-01	NA	NA	cis
cg07156336	AD	2	4.25E-02	8.65E-01	NA	NA	NA	NA	NA	NA	cis
cg07167185	AD	2	-8.79E-02	1.11E-01	NA	NA	NA	NA	NA	NA	cis
cg07313882	AD	2	1.73E-02	5.98E-01	NA	NA	NA	NA	NA	NA	cis
cg07387383	AD	1	NA	NA	NA	NA	NA	NA	-1.87E-02	5.85E-01	cis
cg07422042	AD	5	-1.88E-02	3.26E-01	2.68E-02	8.80E-01	-2.05E-02	3.45E-01	NA	NA	cis
cg08122652	AD	1	NA	NA	NA	NA	NA	NA	-5.80E-02	2.51E-01	cis
cg08923349	AD	3	6.78E-02	1.78E-01	3.22E-03	9.88E-01	6.51E-02	2.22E-01	NA	NA	cis
cg09028383	AD	1	NA	NA	NA	NA	NA	NA	-4.21E-02	3.17E-01	cis
cg09577455	AD	3	5.43E-03	9.08E-01	1.68E-01	6.55E-01	2.22E-02	4.93E-01	NA	NA	cis
cg10287137	AD	1	NA	NA	NA	NA	NA	NA	2.19E-02	8.32E-01	cis
cg10636246	AD	3	-5.70E-02	2.31E-01	4.61E-02	6.81E-01	-4.26E-02	3.85E-01	NA	NA	cis
cg10652637	AD	3	2.93E-02	3.21E-01	-1.78E-01	4.89E-01	3.37E-02	3.13E-01	NA	NA	cis
cg10778971	AD	1	NA	NA	NA	NA	NA	NA	-5.75E-02	3.74E-01	cis
cg11681597	AD	1	NA	NA	NA	NA	NA	NA	-5.30E-02	2.28E-01	cis
cg12200124	AD	2	2.10E-02	8.32E-01	NA	NA	NA	NA	NA	NA	cis
cg12226453	AD	1	NA	NA	NA	NA	NA	NA	-7.63E-02	3.53E-01	cis
cg12359279	AD	1	NA	NA	NA	NA	NA	NA	-1.24E-01	2.51E-01	cis

cg12885484	AD	8	-3.30E-02	1.18E-01	-6.88E-02	2.40E-01	-3.23E-02	1.47E-01	NA	NA	cis
cg12906975	AD	1	NA	NA	NA	NA	NA	NA	-8.96E-03	8.44E-01	cis
cg14064762	AD	3	1.14E-01	7.87E-02	-9.11E-02	8.53E-01	8.83E-02	1.08E-01	NA	NA	cis
cg14106933	AD	6	-5.36E-03	8.12E-01	-2.99E-03	9.63E-01	-1.03E-02	6.93E-01	NA	NA	cis
cg15467116	AD	1	NA	NA	NA	NA	NA	NA	-2.76E-02	3.17E-01	cis
cg15551881	AD	3	7.97E-02	2.73E-01	-1.76E-01	3.78E-01	4.80E-02	3.60E-01	NA	NA	cis
cg16163382	AD	1	NA	NA	NA	NA	NA	NA	2.53E-02	7.92E-01	cis
cg16399664	AD	1	NA	NA	NA	NA	NA	NA	1.83E-01	2.43E-01	cis
cg16734795	AD	2	5.90E-02	3.59E-01	NA	NA	NA	NA	NA	NA	cis
cg16785077	AD	1	NA	NA	NA	NA	NA	NA	-8.00E-02	1.47E-01	cis
cg16871561	AD	1	NA	NA	NA	NA	NA	NA	3.79E-02	7.39E-01	cis
cg16995606	AD	2	-1.50E-02	8.24E-01	NA	NA	NA	NA	NA	NA	cis
cg17021225	AD	4	-5.83E-02	6.76E-02	-4.83E-02	4.93E-01	-6.22E-02	6.91E-02	NA	NA	cis
cg17607231	AD	4	-4.04E-03	8.35E-01	-2.16E-02	5.28E-01	-5.89E-03	7.71E-01	NA	NA	cis
cg17782974	AD	2	5.00E-02	7.95E-01	NA	NA	NA	NA	NA	NA	cis
cg17915189	AD	2	2.99E-02	4.69E-01	NA	NA	NA	NA	NA	NA	cis
cg18101225	AD	1	NA	NA	NA	NA	NA	NA	-3.86E-02	3.69E-01	cis
cg18194850	AD	2	4.38E-02	5.84E-01	NA	NA	NA	NA	NA	NA	cis

cg18587465	AD	1	NA	NA	NA	NA	NA	NA	-5.14E-02	5.91E-01	cis
cg18686270	AD	6	1.06E-03	9.53E-01	-2.21E-02	5.30E-01	-5.30E-03	7.77E-01	NA	NA	cis
cg18805457	AD	1	NA	NA	NA	NA	NA	NA	2.10E-01	1.08E-01	cis
cg19323951	AD	2	-1.08E-02	7.30E-01	NA	NA	NA	NA	NA	NA	cis
cg19371652	AD	1	NA	NA	NA	NA	NA	NA	3.99E-02	5.49E-01	cis
cg19867107	AD	2	1.59E-02	8.46E-01	NA	NA	NA	NA	NA	NA	cis
cg20061654	AD	2	6.59E-03	8.60E-01	NA	NA	NA	NA	NA	NA	cis
cg20566766	AD	1	NA	NA	NA	NA	NA	NA	1.04E-01	2.67E-01	cis
cg20586531	AD	1	NA	NA	NA	NA	NA	NA	-1.48E-02	8.77E-01	cis
cg20808080	AD	1	NA	NA	NA	NA	NA	NA	4.80E-03	9.69E-01	cis
cg21320768	AD	2	5.81E-02	7.40E-02	NA	NA	NA	NA	NA	NA	cis
cg22488164	AD	1	NA	NA	NA	NA	NA	NA	-6.28E-02	2.62E-01	cis
cg22647738	AD	2	-1.08E-02	8.56E-01	NA	NA	NA	NA	NA	NA	cis
cg22737154	AD	1	NA	NA	NA	NA	NA	NA	1.79E-01	1.02E-01	cis
cg22862003	AD	2	6.30E-02	2.44E-01	NA	NA	NA	NA	NA	NA	cis
cg22930808	AD	1	NA	NA	NA	NA	NA	NA	-8.45E-02	2.65E-01	cis
cg23540819	AD	3	-2.63E-02	6.02E-01	-8.38E-02	3.84E-01	-1.96E-02	5.99E-01	NA	NA	cis
cg23642826	AD	1	NA	NA	NA	NA	NA	NA	-6.48E-02	7.07E-01	cis

cg23856536	AD	2	7.80E-02	2.34E-01	NA	NA	NA	NA	NA	NA	cis
cg23930334	AD	4	-5.69E-03	8.26E-01	-2.12E-02	7.54E-01	-7.57E-03	7.86E-01	NA	NA	cis
cg24002003	AD	1	NA	NA	NA	NA	NA	NA	-1.91E-02	7.44E-01	cis
cg24375627	AD	1	NA	NA	NA	NA	NA	NA	-4.35E-02	4.89E-01	cis
cg24430754	AD	1	NA	NA	NA	NA	NA	NA	2.92E-02	4.10E-01	cis
cg24579218	AD	12	9.06E-03	6.28E-01	-2.01E-02	5.67E-01	-8.92E-03	6.09E-01	NA	NA	cis
cg24621362	AD	1	NA	NA	NA	NA	NA	NA	5.90E-03	9.27E-01	cis
cg24819835	AD	1	NA	NA	NA	NA	NA	NA	-2.65E-02	7.70E-01	cis
cg25800166	AD	1	NA	NA	NA	NA	NA	NA	1.76E-01	1.38E-01	cis
cg25800638	AD	1	NA	NA	NA	NA	NA	NA	1.57E-02	8.16E-01	cis
cg26405097	AD	1	NA	NA	NA	NA	NA	NA	5.52E-03	9.37E-01	cis
cg26572165	AD	1	NA	NA	NA	NA	NA	NA	-6.62E-02	4.99E-01	cis
cg26918935	AD	1	NA	NA	NA	NA	NA	NA	7.75E-03	8.98E-01	cis
cg26931488	AD	2	-1.84E-02	6.62E-01	NA	NA	NA	NA	NA	NA	cis
cg27020362	AD	2	6.46E-02	1.74E-01	NA	NA	NA	NA	NA	NA	cis
cg27615938	AD	1	NA	NA	NA	NA	NA	NA	2.87E-02	4.42E-01	cis
cg10636246	AD	1	NA	NA	NA	NA	NA	NA	-3.79E-01	2.74E-06	trans
cg22488164	AD	1	NA	NA	NA	NA	NA	NA	1.57E-01	3.71E-02	trans

cg23642826	AD	2	2.00E-01	5.24E-03	NA	NA	NA	NA	NA	NA	trans
cg24002003	AD	3	7.55E-02	2.58E-02	-2.98E-02	7.96E-01	6.39E-02	7.94E-02	NA	NA	trans
cg00421164	AD	1	NA	NA	NA	NA	NA	NA	-5.76E-02	3.21E-01	trans
cg00607627	AD	2	2.33E-02	5.11E-01	NA	NA	NA	NA	NA	NA	trans
cg02017323	AD	1	NA	NA	NA	NA	NA	NA	2.69E-02	7.66E-01	trans
cg02464912	AD	1	NA	NA	NA	NA	NA	NA	3.69E-02	3.69E-01	trans
cg02560388	AD	1	NA	NA	NA	NA	NA	NA	4.10E-02	6.00E-01	trans
cg02650017	AD	2	7.24E-02	2.13E-01	NA	NA	NA	NA	NA	NA	trans
cg03699074	AD	2	4.81E-02	2.08E-01	NA	NA	NA	NA	NA	NA	trans
cg03781224	AD	1	NA	NA	NA	NA	NA	NA	8.12E-02	2.05E-01	trans
cg03887528	AD	2	5.91E-02	3.20E-01	NA	NA	NA	NA	NA	NA	trans
cg04514047	AD	3	5.73E-03	8.96E-01	-1.70E-02	9.15E-01	6.94E-03	8.78E-01	NA	NA	trans
cg04543273	AD	1	NA	NA	NA	NA	NA	NA	8.92E-02	4.14E-01	trans
cg05573654	AD	3	-1.11E-02	7.21E-01	6.72E-02	5.09E-01	-6.92E-03	8.18E-01	NA	NA	trans
cg06130714	AD	2	-4.74E-02	5.45E-01	NA	NA	NA	NA	NA	NA	trans
cg06679990	AD	1	NA	NA	NA	NA	NA	NA	-5.19E-02	3.83E-01	trans
cg06703222	AD	1	NA	NA	NA	NA	NA	NA	8.94E-02	2.05E-01	trans
cg07387383	AD	1	NA	NA	NA	NA	NA	NA	1.79E-02	8.40E-01	trans

cg07904865	AD	2	-1.54E-01	5.42E-02	NA	NA	NA	NA	NA	NA	trans
cg08156775	AD	1	NA	NA	NA	NA	NA	NA	7.25E-02	4.00E-01	trans
cg08287334	AD	1	NA	NA	NA	NA	NA	NA	-3.34E-02	5.42E-01	trans
cg08525314	AD	3	7.32E-03	8.25E-01	8.86E-02	3.78E-01	7.38E-03	8.09E-01	NA	NA	trans
cg08926253	AD	3	4.04E-02	3.87E-01	-7.52E-02	9.32E-01	4.85E-02	3.41E-01	NA	NA	trans
cg09577455	AD	3	8.00E-02	3.77E-01	-6.78E-01	4.10E-01	8.35E-02	4.01E-01	NA	NA	trans
cg09674502	AD	1	NA	NA	NA	NA	NA	NA	1.31E-01	2.28E-01	trans
cg11681597	AD	1	NA	NA	NA	NA	NA	NA	-2.89E-02	7.63E-01	trans
cg11791770	AD	1	NA	NA	NA	NA	NA	NA	3.15E-02	5.34E-01	trans
cg12200124	AD	1	NA	NA	NA	NA	NA	NA	9.57E-02	2.46E-01	trans
cg12226453	AD	1	NA	NA	NA	NA	NA	NA	-7.47E-02	1.98E-01	trans
cg13030582	AD	1	NA	NA	NA	NA	NA	NA	2.03E-01	8.13E-02	trans
cg14293575	AD	1	NA	NA	NA	NA	NA	NA	-7.77E-02	5.54E-01	trans
cg16113793	AD	1	NA	NA	NA	NA	NA	NA	9.97E-02	3.06E-01	trans
cg16462073	AD	2	3.28E-02	4.75E-01	NA	NA	NA	NA	NA	NA	trans
cg16734795	AD	1	NA	NA	NA	NA	NA	NA	-6.27E-02	4.03E-01	trans
cg17114584	AD	4	-2.00E-02	5.07E-01	-8.57E-02	5.27E-01	9.49E-03	7.26E-01	NA	NA	trans
cg17607231	AD	1	NA	NA	NA	NA	NA	NA	9.31E-02	2.07E-01	trans

cg17915189	AD	1	NA	NA	NA	NA	NA	NA	2.43E-02	7.46E-01	trans
cg18101225	AD	2	5.64E-02	1.33E-01	NA	NA	NA	NA	NA	NA	trans
cg18731055	AD	1	NA	NA	NA	NA	NA	NA	6.94E-02	3.14E-01	trans
cg18805457	AD	1	NA	NA	NA	NA	NA	NA	-7.22E-02	3.21E-01	trans
cg20692268	AD	3	5.63E-02	2.09E-01	2.01E-01	4.60E-01	7.15E-02	1.56E-01	NA	NA	trans
cg22185977	AD	6	-9.77E-03	6.33E-01	-1.76E-02	6.73E-01	-1.10E-02	6.27E-01	NA	NA	trans
cg22737154	AD	1	NA	NA	NA	NA	NA	NA	-2.47E-01	6.25E-02	trans
cg23856536	AD	1	NA	NA	NA	NA	NA	NA	-2.06E-02	8.41E-01	trans
cg24760467	AD	1	NA	NA	NA	NA	NA	NA	2.19E-02	7.66E-01	trans
cg25800638	AD	1	NA	NA	NA	NA	NA	NA	-4.54E-03	9.37E-01	trans
cg27020362	AD	2	5.77E-02	1.34E-01	NA	NA	NA	NA	NA	NA	trans

Table S3: Sensitivity analysis results of MR between 172 CpGs associated with COVID-19 infection on AD.

exp	out	nsnp	het_IVW_P	het_egger_p	pleio_egger_P	pleio_presso_P	r2_exp	F_value	steiger_test_P	type
cg02650017	AD	1	NA	NA	NA	NA	4.3E-03	1.2E+02	6.3E-24	cis
cg04543273	AD	1	NA	NA	NA	NA	8.5E-03	2.3E+02	1.8E-45	cis
cg05411199	AD	1	NA	NA	NA	NA	3.4E-02	9.3E+02	4.3E-191	cis
cg07477602	AD	3	6.5E-01	7.8E-01	5.4E-01	NA	7.6E-02	5.3E+02	0.0E+00	cis
cg08398132	AD	2	3.3E-01	NA	NA	NA	3.7E-03	4.6E+01	1.5E-19	cis
cg09322555	AD	4	3.0E-02	1.2E-02	9.9E-01	3.2E-01	6.9E-02	4.0E+02	0.0E+00	cis
cg11916609	AD	2	9.6E-01	NA	NA	NA	4.7E-02	3.1E+02	1.1E-194	cis
cg20692268	AD	1	NA	NA	NA	NA	4.1E-02	1.1E+03	1.0E-233	cis
cg21549285	AD	6	1.2E-01	2.7E-01	1.8E-01	3.1E-01	7.1E-02	2.0E+02	0.0E+00	cis
cg24772388	AD	2	5.2E-01	NA	NA	NA	1.8E-02	1.4E+02	2.0E-78	cis
cg26312951	AD	1	NA	NA	NA	NA	7.3E-03	1.9E+02	2.4E-38	cis
cg00343127	AD	1	NA	NA	NA	NA	2.9E-02	8.3E+02	5.7E-173	cis
cg00388871	AD	3	8.2E-01	9.8E-01	6.4E-01	NA	2.3E-02	1.6E+02	7.8E-111	cis
cg00421164	AD	1	NA	NA	NA	NA	3.2E-02	8.1E+02	1.4E-171	cis
cg00533183	AD	2	3.6E-03	NA	NA	NA	3.1E-02	3.6E+02	1.2E-150	cis
cg00590152	AD	4	7.3E-01	9.2E-01	4.0E-01	7.1E-01	6.4E-02	1.7E+02	3.4E-207	cis
cg00598235	AD	2	2.0E-01	NA	NA	NA	4.6E-02	5.0E+02	9.5E-225	cis
cg01004980	AD	1	NA	NA	NA	NA	2.2E-03	5.9E+01	1.2E-13	cis
cg01028142	AD	1	NA	NA	NA	NA	4.9E-03	1.3E+02	4.1E-30	cis
cg01459052	AD	1	NA	NA	NA	NA	2.9E-02	8.3E+02	4.9E-173	cis
cg02364826	AD	3	7.7E-01	5.5E-01	7.6E-01	NA	1.1E-01	1.1E+03	0.0E+00	cis
cg02481950	AD	3	7.9E-01	6.8E-01	6.8E-01	NA	5.8E-02	4.3E+02	7.8E-291	cis
cg02583484	AD	2	3.7E-01	NA	NA	NA	2.4E-02	1.9E+02	2.2E-92	cis
cg02741985	AD	5	8.5E-01	7.4E-01	7.3E-01	9.1E-01	1.0E-01	2.8E+02	0.0E+00	cis
cg02744705	AD	2	3.4E-02	NA	NA	NA	4.8E-02	6.3E+02	5.1E-257	cis
cg03234777	AD	5	6.7E-01	5.4E-01	7.1E-01	7.2E-01	1.5E-01	8.1E+02	0.0E+00	cis
cg03290827	AD	4	7.3E-01	9.3E-01	3.9E-01	8.1E-01	9.4E-02	1.4E+02	0.0E+00	cis
cg03398461	AD	1	NA	NA	NA	NA	1.3E-02	3.3E+02	1.8E-70	cis
cg03663120	AD	9	2.4E-01	2.0E-01	5.8E-01	2.9E-01	1.3E-01	1.6E+02	0.0E+00	cis
cg03699074	AD	1	NA	NA	NA	NA	1.4E-02	3.3E+02	2.9E-71	cis
cg03753191	AD	2	4.0E-01	NA	NA	NA	8.5E-03	1.1E+02	8.0E-48	cis
cg03781224	AD	1	NA	NA	NA	NA	3.2E-02	7.4E+02	3.0E-153	cis
cg03887528	AD	2	3.2E-01	NA	NA	NA	1.0E-01	1.1E+03	0.0E+00	cis
cg04248312	AD	5	2.2E-01	1.6E-01	5.9E-01	3.6E-01	2.9E-01	1.8E+03	0.0E+00	cis

cg04306926	AD	1	NA	NA	NA	NA	1.8E-02	5.1E+02	6.2E-107	cis
cg04332373	AD	1	NA	NA	NA	NA	7.7E-03	2.1E+02	8.9E-46	cis
cg04445427	AD	4	8.8E-01	7.9E-01	7.1E-01	8.9E-01	6.9E-02	1.3E+02	0.0E+00	cis
cg04571130	AD	3	3.1E-02	8.4E-02	4.5E-01	NA	2.4E-02	4.1E+01	6.5E-69	cis
cg04577162	AD	2	2.0E-01	NA	NA	NA	1.7E-02	1.9E+02	3.4E-82	cis
cg04871807	AD	5	4.0E-01	4.5E-01	3.2E-01	4.7E-01	2.0E-01	1.3E+03	0.0E+00	cis
cg04955573	AD	4	8.3E-01	9.7E-01	4.6E-01	8.7E-01	1.8E-02	5.0E+01	1.3E-78	cis
cg05316065	AD	1	NA	NA	NA	NA	4.7E-03	1.3E+02	3.2E-29	cis
cg05609160	AD	6	3.7E-01	2.6E-01	7.5E-01	5.7E-01	2.3E-01	2.9E+02	0.0E+00	cis
cg05663031	AD	2	9.2E-01	NA	NA	NA	6.3E-02	7.5E+02	0.0E+00	cis
cg05767306	AD	2	4.3E-01	NA	NA	NA	2.0E-01	3.1E+03	0.0E+00	cis
cg05778424	AD	1	NA	NA	NA	NA	2.4E-02	6.2E+02	3.1E-129	cis
cg05934015	AD	1	NA	NA	NA	NA	6.3E-03	1.8E+02	2.3E-37	cis
cg05955436	AD	6	7.7E-01	7.2E-01	5.3E-01	7.7E-01	1.1E-01	4.6E+02	0.0E+00	cis
cg06043201	AD	1	NA	NA	NA	NA	2.1E-03	5.4E+01	2.1E-12	cis
cg06070445	AD	1	NA	NA	NA	NA	3.0E-02	7.1E+02	1.0E-150	cis
cg06112967	AD	1	NA	NA	NA	NA	8.7E-03	2.4E+02	4.2E-53	cis
cg06146977	AD	2	5.3E-01	NA	NA	NA	1.0E-02	1.2E+02	3.4E-51	cis
cg06850597	AD	1	NA	NA	NA	NA	6.1E-03	1.6E+02	2.4E-34	cis
cg06981309	AD	6	1.0E-01	5.8E-02	9.6E-01	2.2E-01	1.3E-01	2.0E+02	0.0E+00	cis
cg07156336	AD	2	2.2E-03	NA	NA	NA	7.5E-03	7.3E+01	2.5E-36	cis
cg07167185	AD	2	4.3E-01	NA	NA	NA	3.5E-02	9.3E+01	6.0E-111	cis
cg07313882	AD	2	3.7E-01	NA	NA	NA	6.1E-02	5.4E+02	1.3E-259	cis
cg07387383	AD	1	NA	NA	NA	NA	5.0E-02	1.3E+03	3.2E-266	cis
cg07422042	AD	5	9.8E-01	9.5E-01	8.0E-01	9.7E-01	1.5E-01	5.7E+02	0.0E+00	cis
cg08122652	AD	1	NA	NA	NA	NA	1.9E-02	5.3E+02	1.0E-109	cis
cg08923349	AD	3	9.2E-01	9.7E-01	7.6E-01	NA	2.1E-02	1.8E+02	1.1E-117	cis
cg09028383	AD	1	NA	NA	NA	NA	3.1E-02	7.3E+02	3.7E-152	cis
cg09577455	AD	3	5.1E-02	3.6E-02	6.6E-01	NA	8.7E-02	5.6E+02	0.0E+00	cis
cg10287137	AD	1	NA	NA	NA	NA	7.1E-03	1.9E+02	8.9E-41	cis
cg10636246	AD	3	2.9E-01	5.3E-01	3.9E-01	NA	2.6E-02	1.1E+02	1.1E-82	cis
cg10652637	AD	3	4.7E-01	1.0E+00	4.4E-01	NA	6.8E-02	5.6E+02	0.0E+00	cis
cg10778971	AD	1	NA	NA	NA	NA	1.2E-02	3.2E+02	3.7E-67	cis
cg11681597	AD	1	NA	NA	NA	NA	3.3E-02	7.8E+02	4.9E-163	cis
cg12200124	AD	2	1.2E-01	NA	NA	NA	1.4E-02	6.3E+01	1.6E-49	cis
cg12226453	AD	1	NA	NA	NA	NA	7.0E-03	1.1E+02	8.1E-25	cis
cg12359279	AD	1	NA	NA	NA	NA	4.3E-03	1.1E+02	2.6E-24	cis

cg12885484	AD	8	9.8E-02	8.7E-02	4.8E-01	1.5E-01	2.0E-01	3.8E+02	0.0E+00	cis
cg12906975	AD	1	NA	NA	NA	NA	2.5E-02	6.0E+02	2.0E-127	cis
cg14064762	AD	3	1.2E-01	7.2E-02	6.9E-01	NA	2.6E-02	2.4E+02	1.3E-149	cis
cg14106933	AD	6	8.0E-01	6.8E-01	9.7E-01	8.3E-01	9.8E-02	4.5E+02	0.0E+00	cis
cg15467116	AD	1	NA	NA	NA	NA	7.0E-02	1.5E+03	0.0E+00	cis
cg15551881	AD	3	5.0E-02	4.1E-01	2.6E-01	NA	2.9E-02	2.6E+02	7.9E-166	cis
cg16163382	AD	1	NA	NA	NA	NA	5.5E-03	1.5E+02	2.8E-33	cis
cg16399664	AD	1	NA	NA	NA	NA	2.3E-03	6.1E+01	7.0E-14	cis
cg16734795	AD	2	4.4E-01	NA	NA	NA	1.9E-02	7.8E+01	8.0E-40	cis
cg16785077	AD	1	NA	NA	NA	NA	1.8E-02	4.3E+02	5.2E-91	cis
cg16871561	AD	1	NA	NA	NA	NA	4.3E-03	1.1E+02	2.6E-25	cis
cg16995606	AD	2	7.3E-01	NA	NA	NA	1.9E-02	6.1E+01	9.0E-66	cis
cg17021225	AD	4	6.1E-01	4.1E-01	8.6E-01	6.9E-01	5.4E-02	1.2E+02	9.4E-250	cis
cg17607231	AD	4	6.1E-01	5.7E-01	4.9E-01	6.9E-01	1.3E-01	6.8E+02	0.0E+00	cis
cg17782974	AD	2	3.5E-02	NA	NA	NA	6.5E-03	8.8E+01	4.0E-37	cis
cg17915189	AD	2	6.5E-01	NA	NA	NA	3.9E-02	4.6E+02	8.3E-212	cis
cg18101225	AD	1	NA	NA	NA	NA	3.0E-02	8.4E+02	5.2E-174	cis
cg18194850	AD	2	4.4E-01	NA	NA	NA	7.5E-03	6.4E+01	5.4E-36	cis
cg18587465	AD	1	NA	NA	NA	NA	6.8E-03	1.9E+02	2.1E-40	cis
cg18686270	AD	6	6.9E-01	6.7E-01	4.4E-01	6.2E-01	1.4E-01	5.4E+02	0.0E+00	cis
cg18805457	AD	1	NA	NA	NA	NA	2.9E-03	7.6E+01	1.5E-16	cis
cg19323951	AD	2	7.2E-01	NA	NA	NA	5.3E-02	5.0E+02	8.0E-267	cis
cg19371652	AD	1	NA	NA	NA	NA	1.0E-02	2.8E+02	1.8E-59	cis
cg19867107	AD	2	3.2E-01	NA	NA	NA	7.8E-03	9.7E+01	5.5E-42	cis
cg20061654	AD	2	2.6E-01	NA	NA	NA	4.6E-02	2.0E+02	1.0E-172	cis
cg20566766	AD	1	NA	NA	NA	NA	6.0E-03	1.5E+02	2.1E-33	cis
cg20586531	AD	1	NA	NA	NA	NA	7.3E-03	1.8E+02	1.5E-39	cis
cg20808080	AD	1	NA	NA	NA	NA	5.1E-03	3.5E+01	3.8E-09	cis
cg21320768	AD	2	8.8E-01	NA	NA	NA	4.0E-02	5.0E+02	3.1E-220	cis
cg22488164	AD	1	NA	NA	NA	NA	1.5E-02	4.3E+02	3.5E-89	cis
cg22647738	AD	2	8.1E-01	NA	NA	NA	1.1E-02	1.3E+02	1.3E-62	cis
cg22737154	AD	1	NA	NA	NA	NA	4.3E-03	1.2E+02	5.6E-25	cis
cg22862003	AD	2	5.2E-01	NA	NA	NA	1.2E-02	1.2E+02	2.2E-60	cis
cg22930808	AD	1	NA	NA	NA	NA	8.2E-03	2.2E+02	1.6E-47	cis
cg23540819	AD	3	1.3E-01	2.4E-01	3.9E-01	NA	4.5E-02	4.2E+02	3.3E-261	cis
cg23642826	AD	1	NA	NA	NA	NA	1.8E-03	4.5E+01	6.4E-11	cis
cg23856536	AD	2	4.4E-01	NA	NA	NA	1.6E-02	1.7E+02	1.2E-80	cis

cg23930334	AD	4	8.6E-01	7.2E-01	8.0E-01	9.3E-01	7.7E-02	3.8E+02	0.0E+00	cis
cg24002003	AD	1	NA	NA	NA	NA	1.4E-02	3.7E+02	2.1E-78	cis
cg24375627	AD	1	NA	NA	NA	NA	1.3E-02	1.2E+02	1.1E-26	cis
cg24430754	AD	1	NA	NA	NA	NA	4.1E-02	1.0E+03	1.5E-211	cis
cg24579218	AD	12	3.7E-02	4.5E-02	3.3E-01	6.6E-02	3.0E-01	2.4E+02	0.0E+00	cis
cg24621362	AD	1	NA	NA	NA	NA	1.1E-02	1.1E+02	7.2E-25	cis
cg24819835	AD	1	NA	NA	NA	NA	6.3E-03	1.8E+02	9.4E-39	cis
cg25800166	AD	1	NA	NA	NA	NA	4.1E-03	1.1E+02	1.6E-23	cis
cg25800638	AD	1	NA	NA	NA	NA	6.9E-03	1.3E+02	1.8E-29	cis
cg26405097	AD	1	NA	NA	NA	NA	1.0E-02	2.9E+02	8.4E-62	cis
cg26572165	AD	1	NA	NA	NA	NA	5.3E-03	1.5E+02	9.1E-32	cis
cg26918935	AD	1	NA	NA	NA	NA	3.7E-02	2.5E+02	7.7E-55	cis
cg26931488	AD	2	2.2E-01	NA	NA	NA	4.4E-02	5.8E+02	5.6E-250	cis
cg27020362	AD	2	4.9E-01	NA	NA	NA	2.3E-02	2.9E+02	4.8E-126	cis
cg27615938	AD	1	NA	NA	NA	NA	4.4E-02	1.2E+03	1.9E-242	cis
cg10636246	AD	1	NA	NA	NA	NA	8.0E-03	2.2E+02	3.5E-42	trans
cg22488164	AD	1	NA	NA	NA	NA	9.3E-03	2.5E+02	2.8E-52	trans
cg23642826	AD	2	5.7E-01	NA	NA	NA	1.1E-02	1.4E+02	7.5E-60	trans
cg24002003	AD	3	4.5E-01	9.7E-01	4.3E-01	NA	4.3E-02	3.8E+02	6.7E-237	trans
cg00421164	AD	1	NA	NA	NA	NA	1.6E-02	4.1E+02	1.9E-86	trans
cg00607627	AD	2	5.7E-01	NA	NA	NA	4.1E-02	5.8E+02	1.6E-241	trans
cg02017323	AD	1	NA	NA	NA	NA	6.5E-03	1.7E+02	1.1E-37	trans
cg02464912	AD	1	NA	NA	NA	NA	3.3E-02	9.2E+02	3.1E-190	trans
cg02560388	AD	1	NA	NA	NA	NA	8.4E-03	2.2E+02	4.5E-48	trans
cg02650017	AD	2	2.2E-01	NA	NA	NA	2.3E-02	3.3E+02	5.0E-134	trans
cg03699074	AD	2	7.3E-01	NA	NA	NA	3.9E-02	5.5E+02	1.2E-228	trans
cg03781224	AD	1	NA	NA	NA	NA	1.1E-02	3.0E+02	8.7E-63	trans
cg03887528	AD	2	7.1E-01	NA	NA	NA	1.7E-02	2.3E+02	2.4E-97	trans
cg04514047	AD	3	9.1E-01	7.0E-01	8.8E-01	NA	2.7E-02	2.3E+02	6.0E-149	trans
cg04543273	AD	1	NA	NA	NA	NA	3.8E-03	9.6E+01	2.0E-21	trans
cg05573654	AD	3	2.8E-01	3.2E-01	4.3E-01	NA	7.0E-02	3.3E+02	1.8E-283	trans
cg06130714	AD	2	1.0E-01	NA	NA	NA	2.3E-02	2.9E+02	1.9E-123	trans
cg06679990	AD	1	NA	NA	NA	NA	1.5E-02	4.1E+02	2.6E-87	trans
cg06703222	AD	1	NA	NA	NA	NA	9.2E-03	2.4E+02	1.3E-51	trans
cg07387383	AD	1	NA	NA	NA	NA	7.1E-03	1.9E+02	1.2E-41	trans
cg07904865	AD	2	2.8E-01	NA	NA	NA	9.6E-03	1.2E+02	4.1E-53	trans
cg08156775	AD	1	NA	NA	NA	NA	7.0E-03	1.8E+02	4.5E-39	trans

cg08287334	AD	1	NA	NA	NA	NA	1.7E-02	4.4E+02	2.7E-92	trans
cg08525314	AD	3	2.9E-01	8.4E-01	3.6E-01	NA	5.3E-02	4.3E+02	4.6E-280	trans
cg08926253	AD	3	5.7E-01	3.0E-01	9.0E-01	NA	2.5E-02	1.2E+02	2.2E-107	trans
cg09577455	AD	3	2.7E-01	5.7E-01	3.7E-01	NA	1.5E-02	7.9E+01	2.4E-69	trans
cg09674502	AD	1	NA	NA	NA	NA	4.8E-03	1.3E+02	5.0E-28	trans
cg11681597	AD	1	NA	NA	NA	NA	6.5E-03	1.8E+02	1.3E-38	trans
cg11791770	AD	1	NA	NA	NA	NA	2.1E-02	5.7E+02	7.8E-120	trans
cg12200124	AD	1	NA	NA	NA	NA	8.2E-03	2.2E+02	1.9E-47	trans
cg12226453	AD	1	NA	NA	NA	NA	1.4E-02	3.7E+02	1.1E-78	trans
cg13030582	AD	1	NA	NA	NA	NA	3.8E-03	9.8E+01	4.4E-21	trans
cg14293575	AD	1	NA	NA	NA	NA	3.1E-03	8.5E+01	4.3E-19	trans
cg16113793	AD	1	NA	NA	NA	NA	5.9E-03	1.6E+02	4.2E-35	trans
cg16462073	AD	2	3.5E-01	NA	NA	NA	2.5E-02	3.5E+02	1.4E-145	trans
cg16734795	AD	1	NA	NA	NA	NA	7.7E-03	2.1E+02	6.2E-45	trans
cg17114584	AD	4	7.6E-02	5.5E-02	6.0E-01	1.9E-01	1.5E-01	3.2E+02	0.0E+00	trans
cg17607231	AD	1	NA	NA	NA	NA	1.2E-02	2.7E+02	4.5E-58	trans
cg17915189	AD	1	NA	NA	NA	NA	9.9E-03	2.7E+02	1.3E-58	trans
cg18101225	AD	2	7.8E-01	NA	NA	NA	3.5E-02	4.8E+02	2.6E-198	trans
cg18731055	AD	1	NA	NA	NA	NA	1.1E-02	3.1E+02	5.3E-66	trans
cg18805457	AD	1	NA	NA	NA	NA	9.8E-03	2.7E+02	3.3E-58	trans
cg20692268	AD	3	7.0E-01	9.6E-01	5.5E-01	NA	2.6E-02	2.3E+02	1.1E-147	trans
cg22185977	AD	6	9.1E-01	8.3E-01	8.2E-01	9.4E-01	1.3E-01	3.5E+02	0.0E+00	trans
cg22737154	AD	1	NA	NA	NA	NA	5.8E-03	1.5E+02	1.4E-32	trans
cg23856536	AD	1	NA	NA	NA	NA	5.2E-03	1.4E+02	2.5E-31	trans
cg24760467	AD	1	NA	NA	NA	NA	9.7E-03	2.6E+02	4.7E-56	trans
cg25800638	AD	1	NA	NA	NA	NA	1.5E-02	3.5E+02	1.5E-75	trans
cg27020362	AD	2	9.0E-01	NA	NA	NA	3.3E-02	4.6E+02	1.0E-190	trans

Table S4: MR results of CpG related Gene expression(eQTL) on AD.

exp	out	nsnp	IVW	IVW_P	MRegger	MRegger_P	weighted_median	weighted_median_P	Wald_ratio	Wald_ratio_P	resource
IL1RL1	AD	5	-4.3E-01	2.0E-09	-4.1E-01	9.9E-02	-4.3E-01	4.5E-16	NA	NA	eQTLGEN
LMAN2	AD	1	NA	NA	NA	NA	NA	NA	3.8E-01	4.1E-05	eQTLGEN
AIM2	AD	4	-1.5E-02	7.6E-01	1.4E-02	8.6E-01	-1.7E-02	7.4E-01	NA	NA	eQTLGEN
LMAN2	AD	1	NA	NA	NA	NA	NA	NA	4.7E-01	3.9E-04	GTEx
MXD3	AD	1	NA	NA	NA	NA	NA	NA	8.5E-02	3.4E-01	GTEx

Table S5: Sensitivity analysis results of MR between CpG related Gene expression(eQTL) on AD.

exp	out	nsnp	het_IVW_P	het_egger_p	pleio_egger_P	pleio_presso_P	r2_exp	F_value	steiger_test P	resource
IL1RL1	AD	5	4.5E-02	2.1E-02	9.2E-01	3.2E-01	2.7E-02	1.5E+02	9.6E-149	eQTLGEN
LMAN2	AD	1	NA	NA	NA	NA	6.2E-03	1.9E+02	2.1E-37	eQTLGEN
AIM2	AD	4	8.8E-01	8.6E-01	6.0E-01	8.7E-01	2.1E-02	1.3E+02	3.5E-132	eQTLGEN
LMAN2	AD	1	NA	NA	NA	NA	6.0E-02	4.2E+01	2.3E-10	GTEx
MXD3	AD	1	NA	NA	NA	NA	8.5E-02	6.2E+01	1.3E-14	GTEx

Table S6: MR results of CpG on the expression of their related genes.

exp	out	nsnp	IVW	IVW_P	MRegger	MRegger_P	weighted_median	weighted_median_P	Wald_ratio	Wald_ratio_P	type
cg04543273	LMAN2	1	NA	NA	NA	NA	NA	NA	-4.6E-01	2.1E-07	cis
cg04543273	MXD3	1	NA	NA	NA	NA	NA	NA	4.1E-01	1.2E-04	cis
cg10636246	AIM2	3	1.9E-01	5.0E-02	4.3E-01	2.1E-01	2.1E-01	2.9E-03	NA	NA	cis
cg11916609	IL1RL1	2	-1.8E-01	9.9E-02	NA	NA	NA	NA	NA	NA	cis

Table S7: Sensitivity analysis results of MR between CpG on the expression of their related genes.

exp	out	nsnp	het_IVW_P	het_egger_p	pleio_egger_P	pleio_presso_P	r2_exp	F_value	steiger_test_P	type
cg04543273	LMAN2	1	NA	NA	NA	NA	8.5E-03	2.3E+02	6.1E-03	cis
cg04543273	MXD3	1	NA	NA	NA	NA	8.5E-03	2.3E+02	1.5E-01	cis
cg10636246	AIM2	3	1.4E-01	4.9E-01	3.1E-01	NA	2.6E-02	1.1E+02	4.6E-01	cis
cg11916609	IL1RL1	2	6.0E-01	NA	NA	NA	4.7E-02	3.1E+02	9.2E-05	cis
